# Patient-level evaluation of smartwatch-based pain and dehydration detection in nursing home residents: a prospective observational study

**DOI:** 10.3389/fdgth.2026.1930008

**Published:** 2026-09-28

**Authors:** Lorenz Kapral, Fred Bucek, Lisa Lichtenegger, Aylin Albrecht, Oliver Kimberger, Laurenz Berger, Francesco Moscato, Harald Willschke

**Affiliations:** 1 Division of General Anesthesia and Intensive Care Medicine, Department of Anesthesia, Intensive Care Medicine, and Pain Medicine, Medical University of Vienna, Vienna, Austria; 2 Ludwig Boltzmann Institute for Digital Health and Patient Safety, Vienna, Austria; 3 Center for Medical Physics and Biomedical Engineering, Medical University of Vienna, Vienna, Austria; 4 Ludwig Boltzmann Institute for Cardiovascular Research, Vienna, Austria; 5 Austrian Cluster for Tissue Regeneration, Vienna, Austria; 6 Department of Anaesthesia, Emergency Medicine and Intensive Care Medicine, University Hospital Wiener Neustadt—Danube Private University, Wiener Neustadt, Austria

**Keywords:** dehydration, machine learning, nursing home, older adults, pain detection, patient-level evaluation, smartwatch, wearable devices

## Abstract

**Introduction:**

Nursing home residents are at high risk for underrecognized pain and dehydration, particularly those with cognitive impairment who cannot reliably self-report symptoms. Consumer-grade smartwatches capable of continuous physiological monitoring may help detect these conditions, but their feasibility in frail older adults remains underexplored, and evaluation strategies based on random splits of repeated time windows can yield optimistically biased estimates. We therefore evaluated the feasibility of continuous smartwatch monitoring in nursing home residents and explored whether machine learning models using smartwatch-derived data can detect pain and dehydration under patient-independent evaluation.

**Methods:**

In a prospective, observational feasibility study (VIENNESE) conducted at three Caritas nursing homes in Vienna, Austria (February to November 2024), 24 residents were enrolled, 16 began smartwatch monitoring and 13 completed the protocol and contributed sufficient data for analysis (1,414 labelled 120 min windows). Matched XGBoost classifiers were evaluated with nested leave-one-patient-out cross-validation, with hyperparameters tuned inside each fold on training participants only.

**Results:**

Pain detection showed a weak signal, with a mean per-patient AUC of 0.557 (range 0.533 to 0.599 across random seeds) and a pooled window-level AUC of 0.736 (0.662 to 0.784). For dehydration, discrimination remained at chance level (mean per-patient AUC 0.513, range 0.468 to 0.550; pooled 0.484, range 0.393 to 0.593).

**Discussion:**

Continuous smartwatch monitoring was operationally feasible in this population. The exploratory models point to a weak pain-related signal that merits evaluation in larger cohorts with stronger reference standards, whereas hydration status may require different sensing modalities.

## 1 Introduction

The global population aged 65 years and older is projected to double by 2050, with nursing home residents representing one of the most vulnerable and medically complex subgroups (1). These individuals frequently experience undertreated pain and unrecognized dehydration, conditions that significantly impact quality of life, functional status, and clinical outcomes (2, 3).

Pain remains systematically underrecognized and undertreated in nursing homes, particularly among residents with dementia or communication difficulties who cannot self-report symptoms reliably (4). Hadjistavropoulos et al., in an interdisciplinary expert consensus statement, highlighted that 40%–80% of nursing home residents experience chronic pain, yet up to 50% receive inadequate pain management (5). This assessment gap is especially pronounced in non-verbal patients, where Kildal et al. have proposed heart rate (HR) monitoring as a promising approach to identify painful situations in patients unable to notify caregivers (6). Current assessment methods rely on periodic clinical evaluations, typically conducted during scheduled nursing rounds. This intermittent approach may miss critical fluctuations in pain status between assessments, delaying intervention during windows when early treatment could prevent deterioration (7). Continuous monitoring could address this gap by providing physiological data without requiring active patient participation.

Dehydration represents a leading cause of preventable hospitalization among nursing home residents. Teno et al. reported that hospitalizations for infections and dehydration are among the most common acute care events in residents with advanced cognitive impairment (8). Low-intake dehydration is particularly insidious in older adults, as physiological changes with aging—including diminished thirst perception, reduced renal concentrating capacity, and decreased total body water—predispose this population to chronic underhydration (9). Adverse outcomes include cognitive decline, increased infection risk, impaired wound healing, constipation, and mortality (10). However, unlike acute pain, chronic low-intake dehydration develops gradually over days to weeks, and its clinical signs (dry mucous membranes, reduced skin turgor, decreased urine output) may not produce strong, consistent changes in the physiological signals measurable by wrist-worn consumer devices.

Consumer-grade smartwatches have emerged as promising tools for remote health monitoring (11). Modern devices such as the Apple Watch can record HR, heart rate variability (HRV), peripheral oxygen saturation (SpO2), physical activity, and gait-related mobility metrics; however, sampling frequency and availability vary by metric and context, so Apple documentation is used here for feature availability rather than scientific validation (12). Validation studies support Apple Watch HR measurements during laboratory activities, photoplethysmography-derived HRV at population scale, and Apple Health gait parameters in adults and seniors, while also indicating metric-specific limitations (13–15). HRV reflects autonomic nervous system activity and has been associated with pain perception in multiple clinical contexts (16). A meta-analysis by Tracy et al. documented decreased HRV in chronic pain conditions, implicating parasympathetic dysregulation (17). Winslow et al. demonstrated that machine learning algorithms can detect acute pain using physiological signals with promising accuracy (18).

However, two critical gaps remain in the literature. First, nursing home residents remain underrepresented in smartwatch-based health studies. Systematic review evidence on wearable activity tracking in older adults shows that research often focuses on disease and frailty management, is frequently limited to walking-related outcomes, and rarely gives sufficient attention to older adults' lived experience with the technology (19). This gap is particularly significant because nursing home data are inherently difficult to obtain, since residents are medically complex, often cognitively impaired, and not the typical users of smartwatch devices. Demonstrating feasibility in this population is essential before larger validation studies can proceed. Second, many published machine learning studies in clinical settings employ evaluation strategies (such as random splits of time windows) that can produce optimistically biased results by allowing within-patient data leakage. Studies that appear to achieve high accuracy may fail entirely when evaluated on truly unseen patients. Patient-independent evaluation is therefore essential for generating clinically meaningful results.

The primary objective of this study was to evaluate the feasibility of continuous smartwatch monitoring in nursing home residents, a population not typically studied with smartwatch technology. The secondary objective was to explore whether smartwatch-derived physiological and activity data can detect pain and dehydration under patient-independent evaluation.

## 2 Materials and methods

### 2.1 Study design and setting

The VIENNESE study (Vital sIgn wEarables NursiNg homE SEtting) was a prospective, non-interventional feasibility study conducted at three Caritas nursing homes in Vienna, Austria. Participant enrollment occurred between February 15, 2024, and November 4, 2024, with final visits completed by November 28, 2024. The study was approved by the Ethics Committee of the Vienna Hospitals of the Vinzenz Group (EK26/2022) and conducted in accordance with the Declaration of Helsinki. Written informed consent was obtained from all participants.

### 2.2 Participants

Residents were screened for eligibility together with the nursing staff of the participating homes, who know the residents and their daily routines and advised on who would be able to take part. Residents were eligible if they were able to provide informed consent themselves and were willing to wear a smartwatch continuously for four weeks. Residents for whom a legal adult representative (Erwachsenenvertretung) had been appointed, and residents who were bedridden, were not eligible. Further exclusion criteria were the presence of a pacemaker or implantable cardioverter-defibrillator, which the device manufacturer lists as a contraindication to wear, severe dermatological conditions preventing device use, terminal illness with a life expectancy of less than three months, or an inability to comply with study procedures. The target sample size of 40 participants was a pragmatic feasibility target rather than a formal power calculation.

### 2.3 Data collection

All participants received an Apple Watch Ultra 2 (Apple Inc., Cupertino, CA, USA) paired with an iPhone SE 2nd Generation (Apple Inc.) for data collection. This smartwatch was selected because it allowed prolonged wear, offline operation, direct data export, and collection of the physiological and mobility variables required for the study. The watch was worn on the non-dominant wrist continuously (24 h/day, 7 days/week) for 4 weeks, removed only for charging. The Apple Watch continuously measured, calculated, or estimated HR, HRV (calculated as the standard deviation of normal-to-normal intervals, HRV-SDNN), resting HR, SpO2, step count, walking/running distance, walking speed, walking asymmetry, walking double support percentage, flights climbed, stair ascent/descent speed, active and basal energy expenditure, physical effort, stand hours, exercise time, and six-minute walk test distance (when available).

To establish ground truth labels, medically trained study staff conducted comprehensive clinical assessments three times weekly (typically Monday, Wednesday, Friday) between 9:00 and 11:00 AM. Pain was assessed using the visual analog scale (VAS) pain score (0–10). The dehydration assessment incorporated eight distinct clinical indicators: peripheral capillary refill time, skin turgor, mucous membrane status, systolic blood pressure, pulse rate, presence of diarrhea, self-reported thirst, and inferior vena cava (IVC) diameter. Bedside ultrasound of the IVC was performed in the supine position to provide an estimate of intravascular volume.

### 2.4 Feature engineering

Smartwatch data were processed into 120 min non-overlapping time windows aligned with clinical assessments. A comprehensive set of 56 features was derived across four domains, as detailed in Table 1. Time windows lacking HR data for more than 50% of the period were excluded as non-wear periods. Missing continuously measured parameters were imputed using forward-fill limited to 3 windows, followed by patient-specific or global means depending on feature availability. Event-based measurements were imputed with 0. Age and gender were included separately as demographic covariates to capture potential interaction effects with physiological signals.

**Table 1:** Smartwatch-derived features used for model training (56 total).

Domain	Features and aggregations
Vital Signs (26)	HR in bpm (max, median, min, SD), HRV-SDNN in ms (max, median, min), SpO_2_ in % (max, median, min), resting HR in bpm (median), walking HR average in bpm (median), temporal differences in HR and SpO_2_, lag features (1, 3, 6 windows) for HR, HRV, SpO_2_, and resting HR
Activity & Energy (18)	Active/basal energy in kcal (max, median, min, sum), physical effort (max, median, min, sum), step count, distance in meters, flights climbed, stand time/hours, exercise time in minutes, rolling sums (6-window) for energy and physical effort
Gait & Mobility (10)	Walking speed, step length, asymmetry, double support %, stair ascent/descent speed, six-minute walk test distance in meters (all median aggregated)
Demographics (2)	Age, gender

### 2.5 Data quality filter

To ensure reliable evaluation, we applied a data quality filter prior to model training and evaluation. Of the 16 participants who completed the monitoring period, per-patient sample counts varied considerably, reflecting differences in device adherence and monitoring duration. Patients contributing fewer than 30% of the median sample count were excluded from the analysis. This threshold was chosen to exclude participants whose limited data would provide unreliable per-patient evaluation while retaining the maximum number of informative participants.

### 2.6 Label definitions

To train the machine learning models, we converted clinical assessments into binary classification targets. Clinical assessments were recorded with a date, and the resulting label was assigned to the smartwatch windows of that day; days without an assessment were excluded rather than interpolated. Assessments were performed during the morning nursing round, so labelled windows fall around a consistent time of day.

For pain, we used the VAS score directly, without incorporating pain medication status, and analysed it as a binary outcome using a threshold of more than 4 on the 0 to 10 scale. This corresponds to the established cut-point for moderate or greater pain, the level at which analgesic intervention is generally indicated in clinical practice, and it was fixed before any modelling. A sensitivity analysis across neighbouring thresholds is reported in Supplementary Table S2. We chose a purely physiological ground truth because including analgesic use would conflate detection of pain physiology with detection of treatment.

Missing clinical values were uncommon, affecting between 0.6% and 3.8% of the 159 assessments for each variable; a single VAS score was missing, and that assessment carried no date and therefore contributed no time windows. Per-variable counts are given in Supplementary Table S5.

For dehydration, the composite clinical score (see (Table 2)) includes an inferior vena cava criterion that compares each measurement with the participant's mean diameter over the observation period. Missing clinical values were filled with the participant's most frequently recorded value. The dehydration endpoint is reported as exploratory.

**Table 2:** Clinical dehydration scoring system.

Clinical indicator	Score points
Capillary refill time ≥2 s	+5
Abnormal skin turgor	+5
Dry mucous membranes	+5
Self-reported thirst	+5
Presence of diarrhea	+5
IVC diameter < patient mean	+5
Systolic blood pressure ≤90 mmHg	+3
Systolic blood pressure 91–100 mmHg	+2
Systolic blood pressure 101–110 mmHg	+1
Pulse rate >131 bpm	+3
Pulse rate 111–131 bpm	+2
Pulse rate 91–111 bpm	+1

### 2.7 Machine learning models

We employed matched classifiers for both detection tasks. Pain and dehydration detection each used an Extreme Gradient Boosting (XGBoost) classifier within a pipeline consisting of StandardScaler for feature normalization and XGBClassifier.

Hyperparameters were optimised separately within each outer leave-one-patient-out fold, using only the participants in that fold's training set, with a randomised search over the same parameter space for both endpoints. The inner search was grouped by participant, so that windows from one person could not fall on both sides of the inner split. Model selection was therefore patient-independent, as was model fitting. The full analysis pipeline is summarised in Supplementary Figure S4.

### 2.8 Evaluation strategy

A central methodological priority of this study was to estimate performance on patients not used for model fitting. After hyperparameter optimization, we therefore employed leave-one-patient-out cross-validation (LOPOCV) for both endpoints. In each fold, all data from one patient were held out as the test set, while the remaining patients formed the training set. Patients with constant labels across all visits were retained exclusively in the training pool, as a held-out patient-specific AUC cannot be computed when the test labels do not vary.

All data-dependent preprocessing that could carry information between participants is fitted inside each outer fold. Feature scaling and class resampling are steps of the modelling pipeline and are therefore estimated on the training participants of that fold alone. Missing smartwatch features are filled within a participant, by forward filling and then that participant's own mean, and missing clinical values are filled with that participant's modal value. Both the within-participant imputation and the inferior vena cava criterion use a participant's measurements from across the whole observation period.

Held-out performance was assessed using AUC, accuracy, precision, recall, and F1 score. Shapley additive explanations were computed for every held-out fold, each fold's model explaining its own held-out windows, and pooled after normalising each fold by its mean absolute contribution, so that no fold is selected and none dominates. For both endpoints we report pooled held-out metrics across all test predictions and the mean held-out-patient AUC across LOPOCV folds. The pooled metrics summarize overall window-level discrimination, whereas the mean held-out-patient AUC describes performance within individual participants and is the primary endpoint. Confidence intervals for pooled measures were obtained by resampling participants rather than windows.

A per-patient AUC cannot be calculated for participants whose label did not change during follow-up, so the primary analysis is restricted to participants with a changing label. A sensitivity analysis in which all participants serve as outer test folds, contributing to pooled metrics only, is reported in Supplementary Table S3. Because the selected models are strongly regularised, single-run estimates were sensitive to the random seed, and performance is therefore reported as the mean across five random seeds with the observed range.

## 3 Results

### 3.1 Study population and feasibility

More than 100 residents were screened for eligibility, of whom approximately 40 met the inclusion criteria and 24 consented. Residents who were approached but not enrolled declined to sign the participant information sheet, proved bedridden on assessment, or had a pacemaker or implantable cardioverter-defibrillator. Eight residents withdrew consent shortly after enrolment, so 16 began monitoring. Three residents discontinued after 1 to 7 days and were removed by the data-quality filter; the remaining 13 completed the monitoring period and form the analysis cohort (Figure 1). Recruitment could not be extended because the participating homes had been fully screened, and the pragmatic target of 40 participants was therefore not reached.

**Figure 1:**
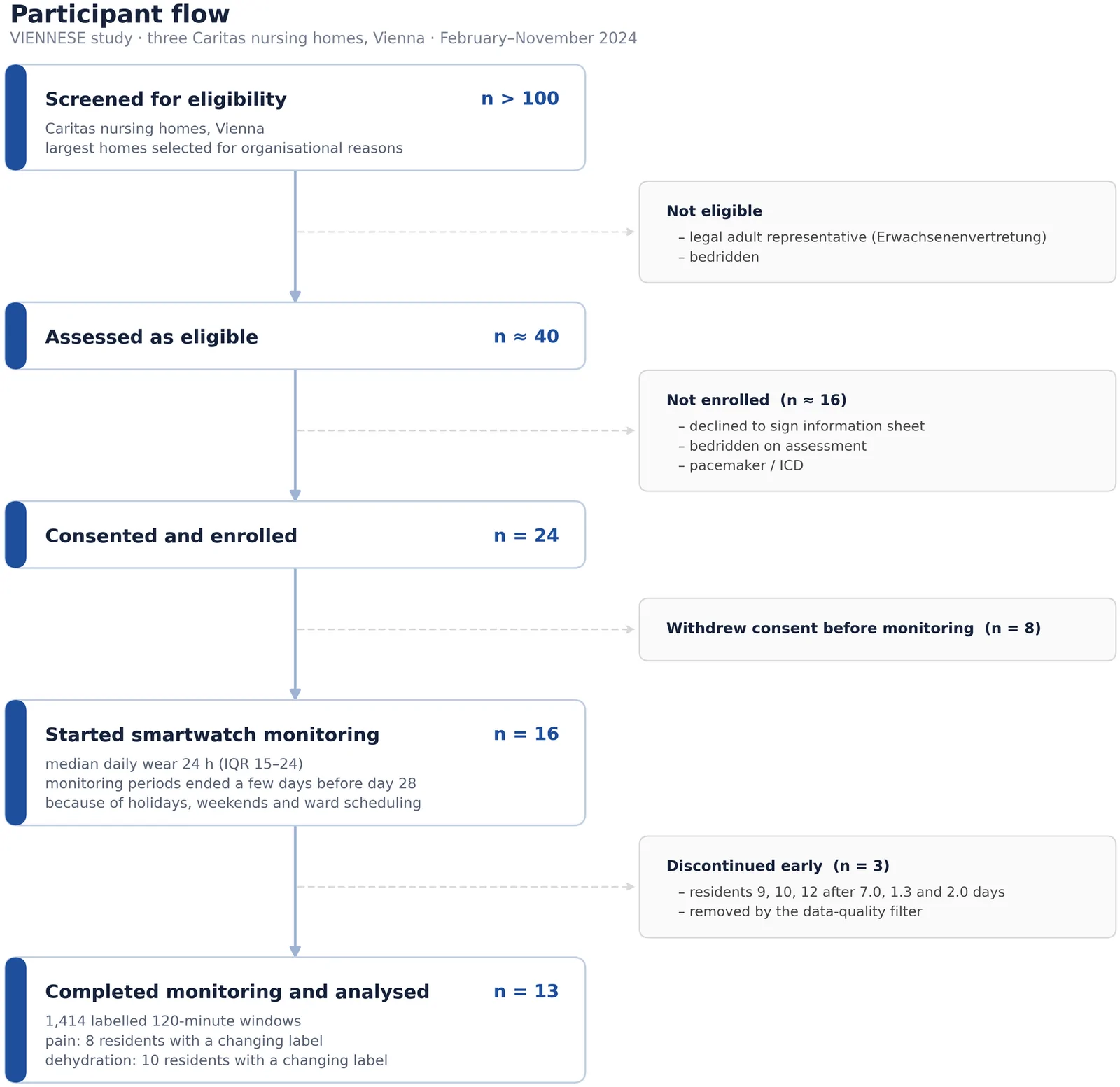
Participant flow in the VIENNESE study. The diagram shows each stage with the reasons recorded at that stage: screening (*n* > 100), eligibility assessment (*n* ≈ 40), enrolment (*n* = 24), start of monitoring (*n* = 16), and the analysis cohort of participants who completed monitoring (*n* = 13 for both tasks). Three participants discontinued after 1 to 7 days and were removed by the participant-level data-quality filter.

Participants who began monitoring had a mean age of 83.4 years (SD 10.7, range 62 to 96), 12 (75.0%) were female, and mean BMI was 30.4 kg/m^2^ (SD 7.3). Six of 16 participants received strong opioid analgesics and 14 of 16 received non-opioid analgesics, most commonly metamizole. No adverse events such as skin irritation, device-related discomfort, or falls attributed to device wear were reported.

Device adherence was high, with a median daily wear time of 24 h (IQR, 15 to 24; mean 20.0 h) and a median monitoring period of 25 days. Monitoring commonly ended a few days before day 28 because of public holidays, weekends and ward scheduling. Before the patient-level data-quality filter the dataset comprised 1,460 labelled windows across 16 participants; the filter removed the three who discontinued early, leaving 1,414 labelled windows across 13 participants for analysis.

Three patients were excluded from both the pain and dehydration analyses based on the data quality filter (IDs 9, 10, 12; contributing 3–30 windows each, fewer than 30% of the median sample count). The final analysis included 13 patients and 1,414 time windows for both tasks.

### 3.2 Label distribution

Figure 2 summarizes the class distribution used for model development. At the assessment level, pain labels comprised 120 negative and 39 positive evaluations (24.5% prevalence). For dehydration, 71 negative and 88 positive assessments (55.3% prevalence) were recorded. After merging clinical labels with 120 min smartwatch time windows, the pain dataset contained 1,050 negative and 364 positive windows (25.7% positive). Using the uncapped dehydration raw-score threshold (raw score >10), the dehydration dataset contained 614 negative and 800 positive windows (56.6% positive).

**Figure 2:**
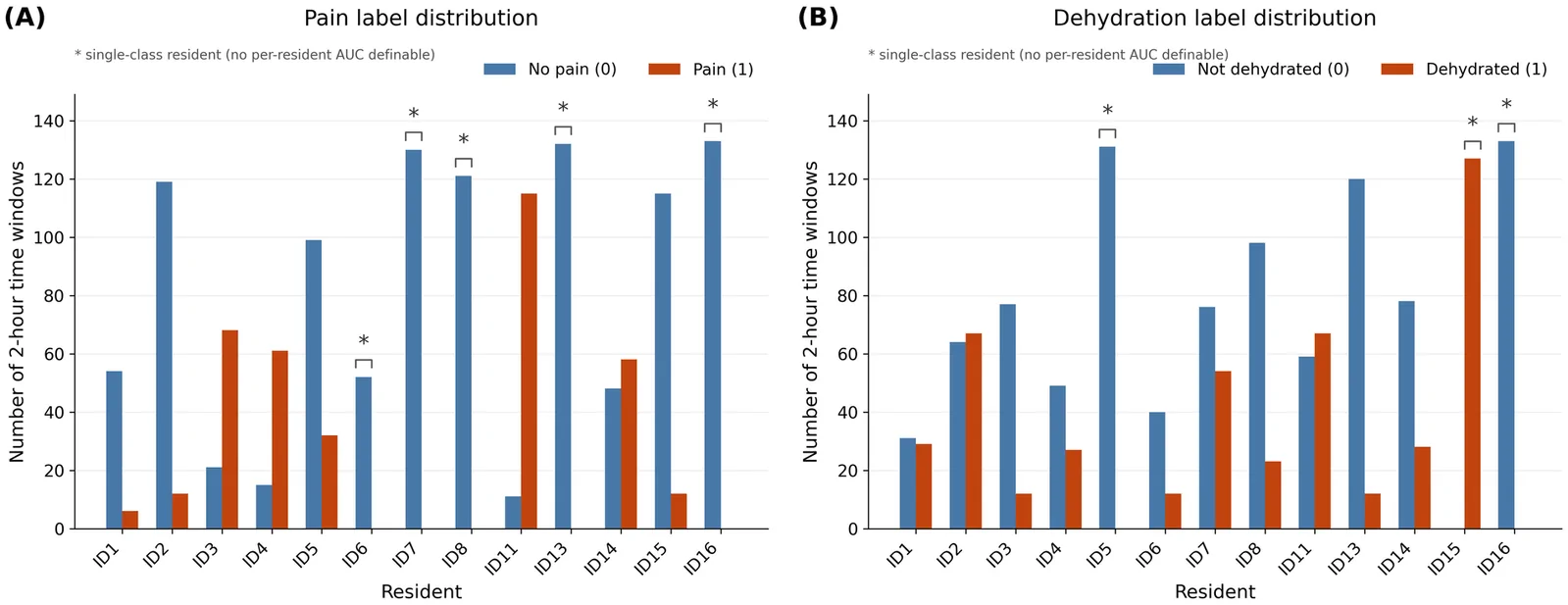
Per-participant label distribution for pain and dehydration after the participant-level quality filter. **(A)** Pain, **(B)** dehydration. Asterisks denote participants with a single class present, for whom a per-participant AUC is undefined; these participants are excluded from the primary held-out evaluation and included in the all-participant sensitivity analysis.

### 3.3 Pain detection

The pain detection model was evaluated using nested LOPOCV. Of the 13 quality-filtered participants, 8 had a changing pain label and were evaluable as held-out folds; the remaining participants contributed to the training set only, and to pooled predictions in the all-participant sensitivity analysis. Across 8 LOPOCV folds (846 held-out windows), the averaged held-out ROC curve reached an AUC of 0.557 (Figure 3A); the internal three-fold cross-validation within the training participants of each fold gave 0.595, and both are reported in Table 3. The mean per-patient AUC was 0.557 (range 0.533 to 0.599 across five random seeds; 95% CI across participants 0.483 to 0.626), with 7 of the 8 evaluable participants scoring above chance, indicating a weak but consistently directed signal. At the pooled window level the model achieved an accuracy of 0.652, precision of 0.613, recall of 0.501 and F1 score of 0.549, with a pooled AUC of 0.736 (Table 3). No VAS score used in the analysis was imputed. Restricting the data to a single prespecified window per clinical assessment, which reduces the number of labelled observations roughly ninefold, gave mean per-patient AUCs of 0.524, 0.582 and 0.655 for the first, midday and last window of the assessment day, against 0.605 using all windows (Supplementary Table S1).

**Figure 3:**
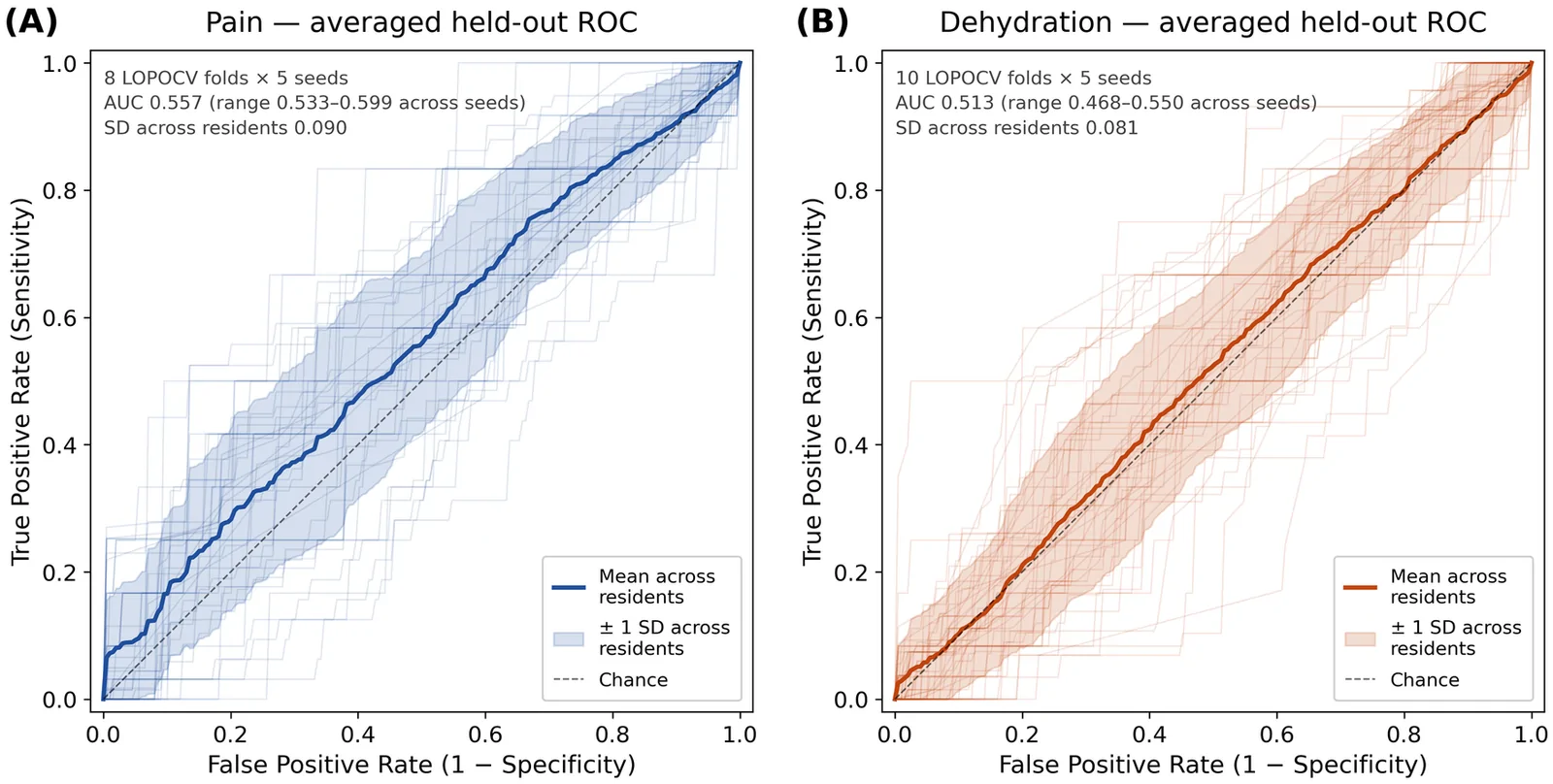
Averaged held-out ROC curves under nested leave-one-patient-out cross-validation. **(A)** Pain detection across the 8 evaluable LOPOCV folds. **(B)** Dehydration detection across the 10 evaluable folds. Curves are averaged over all folds of five random seeds; faint lines show individual folds and the shaded band indicates one standard deviation across participants. Both curves lie close to the chance diagonal.

**Table 3:** Participant demographics.

Characteristic	Value
Initially consented, *n*	24
Completed monitoring, *n*	16
Age, years, mean (SD)	83.4 (10.7)
Age range, years	62–96
Female, *n* (%)	12 (75.0)
BMI, kg/m^2^, mean (SD)	30.4 (7.3)
Monitoring duration, days, planned	28 per participant
Time windows per patient, mean (SD)	91 (46)
Patients in pain analysis, *n*	13
Patients in dehydration analysis, *n*	13

In an exploratory *post hoc* analysis, we performed a gender-stratified pain analysis. The female subgroup (9 participants, 7 evaluable LOPOCV folds) achieved an averaged held-out ROC AUC of 0.571 (range 0.544 to 0.592 across seeds; Supplementary Figure S3). The male subgroup could not be evaluated because only one male participant had a changing pain label, and that held-out fold lacked sufficient class variation.

### 3.4 Dehydration detection

The dehydration detection model used the same XGBoost pipeline but the stricter uncapped raw-score label. Of the 13 quality-filtered participants, 10 had a changing dehydration label and were evaluable as held-out folds. Across 10 LOPOCV folds (1,023 held-out windows), the averaged held-out ROC curve reached an AUC of 0.513 (Figure 3B); the corresponding internal cross-validation estimate was 0.468. The mean per-patient AUC was 0.513 (range 0.468 to 0.550; 95% CI 0.412 to 0.616), with 4 of 10 participants above chance. At the pooled window level accuracy was 0.547, precision 0.287, recall 0.265 and F1 score 0.274, with a pooled AUC of 0.484 (Table 3). The inferior vena cava measurement was not obtained at 67 of the 159 assessments (42%), so for those assessments one of the eight components could not contribute to the score. Restricting the analysis to assessments in which every component was observed retained 806 of 1,414 windows and gave a mean per-patient AUC of 0.535 (range 0.505 to 0.561 across seeds; pooled 0.481), leaving the conclusion unchanged (Supplementary Table S6). A performance comparison of the pain and dehydration models is shown in (Table 4).

**Table 4:** Performance comparison of the pain and dehydration models evaluated by patient-level LOPOCV.

Metric	Pain model	Dehydration model
Discrimination		
Mean per-patient AUC (95% CI)	0.557 (0.483−0.626)	0.513 (0.412−0.616)
Mean per-patient AUC, range across seeds	0.533–0.599	0.468–0.550
Participants scoring above chance	7/8	4/10
AUC, averaged held-out ROC curve (range)	0.557 (0.533–0.599)	0.513 (0.468–0.550)
AUC, internal 3-fold CV within training set (range)	0.595 (0.592–0.598)	0.468 (0.466–0.470)
Pooled window-level AUC (range)	0.736 (0.662–0.784)	0.484 (0.393–0.593)
Pooled window-level metrics (mean, range)		
Accuracy	0.652 (0.603–0.729)	0.547 (0.503–0.580)
Precision	0.613 (0.550–0.683)	0.287 (0.256–0.307)
Recall	0.501 (0.426–0.692)	0.265 (0.239–0.296)
F1 score	0.549 (0.480–0.688)	0.274 (0.268–0.287)
Brier score	0.212 (0.180–0.252)	0.281 (0.245–0.318)
Evaluation details		
Evaluation scheme	nested LOPOCV	nested LOPOCV
Evaluable held-out participants	8	10
Pooled test windows	846	1,023
Training participants per fold	12	12
Classifier	XGBoost	XGBoost

Per-patient LOPOCV metrics for both endpoints are provided in the Supplementary Material.

### 3.5 Feature importance and statistical analysis

We assessed feature importance through two complementary approaches: fold-averaged importance rankings across all LOPOCV folds, and SHAP contributions pooled across every held-out fold (Figure 4). Both therefore describe the full held-out set rather than a selected model. The two detection tasks exhibited distinct feature-importance patterns. For pain detection, the averaged importance profile was dominated by gait and mobility measures led by stair ascent speed, walking asymmetry, double support percentage and walking speed, followed by activity and energy measures.

**Figure 4:**
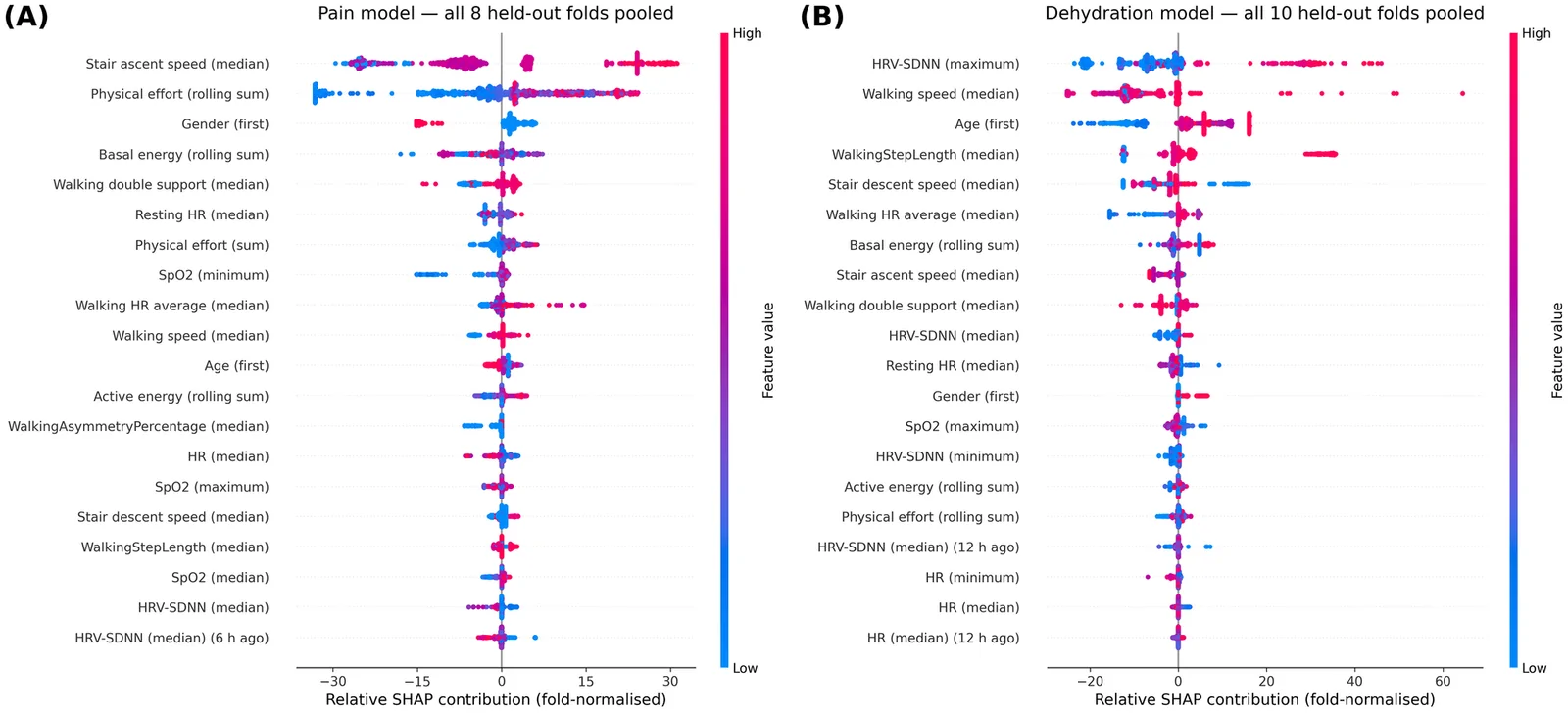
SHAP feature contributions pooled across all held-out folds. Left **(A)**: pain model. Right **(B)**: dehydration model. Each fold's model explains its own held-out windows and the results are combined, so that every held-out observation is represented. Because the folds have separately tuned models whose contributions lie on different scales, each fold's values are normalised by its mean absolute contribution before pooling, and the axis therefore shows relative contribution. Age and gender rank highly for both endpoints despite being constant within a participant.

For dehydration detection, the highest-ranked features shifted toward walking speed and HRV-derived variables, followed by age, stair descent speed, six-minute walk test distance, and walking HR. Feature values in the two label states are reported descriptively in Table 5; inferential tests are not reported, because repeated windows from the same participant are not independent observations.

**Table 5:** Selected top-ranked features in the two label states.

Feature	Mean, negative	Mean, positive
Pain model, selected displayed features		
Stair ascent speed	0.308	0.309
Physical effort (rolling sum)	6,875.938	7,519.847
Walking asymmetry	0.004	0.004
Walking speed	1.088	1.068
Dehydration model, selected displayed features		
Walking speed	1.135	1.136
HRV-SDNN (median)	46.127	48.810
HRV-SDNN (max)	46.181	48.985
HRV-SDNN (min)	46.074	48.636

Values are the mean across participants of each participant's own mean, so that every participant carries equal weight regardless of how many time windows they contributed. Age and gender are not shown, as they cannot differ between label states within a participant.

## 4 Discussion

This prospective feasibility study evaluated continuous smartwatch monitoring and patient-independent machine-learning models for pain and dehydration detection in nursing home residents. It yielded two findings. First, among residents who were screened as able to comply and who completed the protocol, continuous smartwatch wear was operationally feasible, with a median daily wear time of 24 h. Second, under patient-independent evaluation pain showed a weak signal, with a mean per-patient AUC of 0.557 (range 0.533 to 0.599 across random seeds) and a pooled window-level AUC of 0.736 (0.662 to 0.784), while dehydration discrimination remained at chance level (0.513, range 0.468 to 0.550; pooled 0.484, range 0.393 to 0.593).

The pooled and per-patient AUCs summarise the same held-out predictions from different angles: the pooled value describes discrimination across all windows in the cohort, the per-patient value discrimination within an individual. We report both, as each is informative for a different question.

The pain result is compatible with a weak pain-related signal, most plausibly a mixture of pain physiology and correlated mobility or activity patterns, and its direction was consistent across participants. This interpretation is hypothesis-generating. The exploratory female-only analysis gave a mean held-out AUC of 0.571 (range 0.544 to 0.592 across seeds), marginally above the mixed-gender value of 0.557 (0.533 to 0.599); with seven participants and no evaluable male subgroup we draw no inference from this comparison.

For dehydration, tightening the label definition and adopting the same XGBoost pipeline left discrimination at chance level (averaged held-out ROC AUC 0.513, range 0.468 to 0.550 across seeds). Individual dehydration signs are clinically relevant but nonspecific in older adults and can vary with comorbidities, medication, acute illness, and measurement conditions (21). We additionally examined individual dehydration sub-indicators in isolation, but no single clinical sign produced a consistent patient-independent signal from wrist-worn data alone. A more plausible interpretation is that chronic low-intake dehydration in older adults does not produce sufficiently strong or consistent changes in the physiological signals measurable by a consumer wrist-worn device, that the pragmatic clinical label introduced substantial noise, or both. Alternative sensing modalities such as bioimpedance, electrodermal activity, or stronger reference standards may be required to capture hydration-related changes that standard optical sensors cannot detect.

The main finding of this study is a practical one: continuous smartwatch monitoring over four weeks was achievable in a real-world nursing home cohort, a population that is rarely represented in smartwatch research. The algorithmic results are exploratory at this stage. The pain models point to a weak signal that warrants evaluation in larger cohorts with stronger reference standards, while hydration status is likely to require different sensing modalities or more specific labels. These findings support future studies using smartwatches for adherence monitoring, activity and mobility phenotyping, and longitudinal observation of older nursing home residents.

Some limitations should be considered when interpreting these findings. The cohort was small and recruited from three nursing homes in a single city. Eligibility required participants to consent for themselves and to follow the study procedures, so the findings apply to residents who were able to participate independently rather than to those unable to self-report, and recruitment could not be extended once the participating homes had been fully screened.

The pain reference standard is a pragmatic one, chosen to be feasible in routine nursing home care. VAS is a self-report measure and reflects a single moment in a symptom that fluctuates, most participants received analgesic treatment, so the labels describe pain under treatment, and many participants carried chronic painful diagnoses, so the labels combine chronic baseline symptoms with acute episodes. Limitations of the dehydration score are discussed above.

Clinical labels were obtained intermittently, whereas smartwatch data were collected continuously throughout the monitoring period. This temporal mismatch may have introduced label noise, particularly for pain, which can fluctuate over short time scales and may depend on movement, positioning, time of day, and chronic baseline symptoms. A sensitivity analysis restricted to one prespecified window per clinical assessment left the conclusions unchanged (Supplementary Table S1). Future studies should incorporate validated observational pain instruments alongside self-report measures.

Finally, the effective sample size for patient-independent generalisation is the number of participants rather than the number of windows, so estimates from a cohort of this size are necessarily imprecise. We therefore report performance as a distribution across random seeds rather than as a single value, and regard the algorithmic findings as hypothesis-generating. This is, in our view, the appropriate level of claim for a feasibility study of this kind.

The dehydration endpoint was based on a pragmatic composite clinical score rather than a laboratory-confirmed reference standard. The included signs are clinically relevant but nonspecific in frail older adults and may be affected by comorbidities, medications, acute illness, and measurement variability. Pulse and blood pressure are also physiologically related to variables partly represented in smartwatch data, which may introduce label-feature coupling. The limited dehydration performance may therefore reflect the characteristics of wrist-worn smartwatch signals, of the dehydration label, or both. Future studies should include stronger reference standards such as serum osmolality, sodium, urine parameters, fluid-balance data, or standardized clinical adjudication, and may require additional sensing modalities such as bioimpedance or electrodermal activity.

Finally, repeated time windows from the same participant were temporally correlated. Although LOPOCV was used to prevent patient-level data leakage, window-level metrics should be interpreted with that structure in mind, and feature comparisons are therefore reported with the participant as the unit of analysis. To make the spread between participants explicit, Supplementary Figures S1, S2 show the held-out ROC curve for every evaluated participant alongside the mean, so that the variability underlying the fold-averaged results in Figure 3 is visible rather than summarised away. The cohort was predominantly female, limiting subgroup analyses and raising the possibility that demographic variables partly captured between-participant differences rather than within-participant physiological responses. Larger, more diverse, externally validated cohorts are needed before clinical translation.

In conclusion, continuous smartwatch monitoring was operationally feasible in nursing home residents who were screened as able to comply and who completed the protocol, with a median daily wear time of 24 h. Under patient-independent evaluation pain detection retained a weak signal (mean per-patient AUC 0.557, range 0.533 to 0.599 across seeds; pooled 0.736, range 0.662 to 0.784), while dehydration discrimination remained at chance level (0.513, range 0.468 to 0.550; pooled 0.484, range 0.393 to 0.593). The principal contribution of this study is therefore practical rather than algorithmic: it demonstrates that prolonged consumer smartwatch monitoring can be carried out in a real-world nursing home cohort, a population rarely represented in wearable research. Larger and more representative cohorts, stronger and preferably observational clinical reference standards, and alternative sensing modalities for hydration are required before clinical translation can be considered.

## Data Availability

The analysis code generated for this study is publicly available on GitHub (20). The datasets presented in this article are not readily available due to privacy regulations and the sensitive nature of health data from nursing home residents. Aggregate outputs may be made available upon reasonable request to the corresponding author, subject to ethics committee approval. Requests to access the datasets should be directed to Lorenz Kapral, lorenz.kapral@meduniwien.ac.at.
