# Supplementary material for: Patient-level evaluation of smartwatch-based pain and dehydration detection in nursing home residents: a prospective observational study
Source: Front Digit Health. 2026 Sep 28;8:1930008. doi: 10.3389/fdgth.2026.1930008 (PMC13654990; doi:10.3389/fdgth.2026.1930008)
Supplement: Supplementary file 1 [file Datasheet1.docx]

# Supplementary Material

*Patient-Level Evaluation of Smartwatch-Based Pain and Dehydration Detection in Nursing Home Residents: A Prospective Observational Study*

## Supplementary Table S1. Label mapping sensitivity analysis

In the primary analysis the label from a clinical assessment is assigned to the 120-minute windows of the day on which the assessment was made. To test whether this affects the results, the evaluation was repeated using a single prespecified window per assessment under three rules fixed in advance: the first window of the assessment day, the window covering midday, and the last window of the day. Evaluation was otherwise identical to the primary analysis.

| **Endpoint** | **Label mapping** | **Observations** | **Mean per-participant AUC** | **Pooled AUC** |
| --- | --- | --- | --- | --- |
| Pain | all windows (primary) | 846 | 0.605 | 0.654 |
| Pain | one per assessment, first | 91 | 0.524 | 0.585 |
| Pain | one per assessment, midday | 91 | 0.582 | 0.525 |
| Pain | one per assessment, last | 91 | 0.655 | 0.560 |
| Dehydration | all windows (primary) | 1023 | 0.541 | 0.477 |
| Dehydration | one per assessment, first | 109 | 0.483 | 0.403 |
| Dehydration | one per assessment, midday | 109 | 0.500 | 0.416 |
| Dehydration | one per assessment, last | 109 | 0.611 | 0.502 |

Restricting the data to one window per assessment reduces the number of labelled observations roughly ninefold. The conclusions are unchanged under all three rules, and the variation between rules is of the same order as the variation between random seeds reported in the main text.

## Supplementary Table S2. Pain threshold sensitivity analysis

Pain was analysed as a binary outcome with the cut-point fixed in advance at the midpoint of the 0 to 10 VAS scale. The table reports the evaluation repeated at the two neighbouring thresholds. Values were computed under the original tuning configuration, since the purpose of this analysis is the comparison across thresholds rather than the absolute level of performance.

| **Threshold** | **Windows** | **Positive windows** | **Participants with a changing label** | **Mean per-participant AUC** | **Pooled AUC** |
| --- | --- | --- | --- | --- | --- |
| VAS > 3 | 1414 | 450 | 6 | 0.547 | 0.594 |
| VAS > 4 | 1414 | 364 | 8 | 0.555 | 0.657 |
| VAS > 5 | 1414 | 174 | 6 | 0.550 | 0.454 |

The mean per-participant AUC is essentially flat across the three thresholds, so the conclusions do not depend on where the cut-point was placed. The chosen threshold also yields the largest number of participants with a changing label, and therefore the largest number of evaluable held-out folds.

## Supplementary Table S3. Analysis including constant-label participants

A per-participant AUC cannot be defined for a participant whose label does not change during follow-up, so the primary analysis is restricted to participants with a changing label. In this analysis every participant serves as an outer test fold; the constant-label participants contribute to the pooled metrics only. Values are the mean across five random seeds.

| **Endpoint** | **Test folds** | **n folds** | **Windows** | **Pooled AUC** | **Accuracy** | **Precision** | **Recall** |
| --- | --- | --- | --- | --- | --- | --- | --- |
| Pain | participants with a changing label | 8 | 846 | 0.736 | 0.652 | 0.613 | 0.501 |
| Pain | all participants | 13 | 1414 | 0.682 | 0.669 | 0.386 | 0.501 |
| Dehydration | participants with a changing label | 10 | 1023 | 0.484 | 0.547 | 0.287 | 0.265 |
| Dehydration | all participants | 13 | 1414 | 0.429 | 0.477 | 0.219 | 0.241 |

Including participants whose label did not change lowers pooled discrimination and precision for both endpoints, because these participants contribute large numbers of single-class windows on which the model produces false positives. The restriction used in the primary analysis is therefore conservative with respect to the reported figures.

## Supplementary Table S4. Per-participant held-out performance

Held-out AUC for each evaluable participant under nested LOPOCV, averaged across five random seeds, together with the number of positive and negative windows that participant contributed. Participants whose label did not change during follow-up are not listed, as a per-participant AUC is not defined for them.

| **Endpoint** | **Participant** | **Held-out AUC** | **Positive windows** | **Negative windows** |
| --- | --- | --- | --- | --- |
| Pain | 1 | 0.601 | 6 | 54 |
| Pain | 2 | 0.642 | 12 | 119 |
| Pain | 3 | 0.507 | 68 | 21 |
| Pain | 4 | 0.606 | 61 | 15 |
| Pain | 5 | 0.401 | 32 | 99 |
| Pain | 11 | 0.609 | 115 | 11 |
| Pain | 14 | 0.582 | 58 | 48 |
| Pain | 15 | 0.507 | 12 | 115 |
| Pain | mean | 0.557 |  |  |
| Dehydration | 1 | 0.430 | 29 | 31 |
| Dehydration | 2 | 0.513 | 67 | 64 |
| Dehydration | 3 | 0.461 | 12 | 77 |
| Dehydration | 4 | 0.524 | 27 | 49 |
| Dehydration | 6 | 0.588 | 12 | 40 |
| Dehydration | 7 | 0.522 | 54 | 76 |
| Dehydration | 8 | 0.512 | 23 | 98 |
| Dehydration | 11 | 0.601 | 67 | 59 |
| Dehydration | 13 | 0.528 | 12 | 120 |
| Dehydration | 14 | 0.452 | 28 | 78 |
| Dehydration | mean | 0.513 |  |  |

## Supplementary Table S5. Missing clinical data

Counts of assessments in which each clinical variable was not recorded. Values used in the models were filled with that participant's own modal value; no value was derived from another participant. The inferior vena cava measurement is listed separately because it was not imputed: where it is absent, that component simply contributes no points to the composite score.

| **Clinical variable** | **Recorded** | **Missing** | **Missing (%)** |
| --- | --- | --- | --- |
| vas score | 158 | 1 | 0.6 |
| pulse 1 min | 156 | 3 | 1.9 |
| blood pressure mmhg | 155 | 4 | 2.5 |
| blood pressure diastolic m | 153 | 6 | 3.8 |
| diarrhea | 156 | 3 | 1.9 |
| dry muscous membranes | 156 | 3 | 1.9 |
| is the skin turgor normal | 156 | 3 | 1.9 |
| self reported thirst | 156 | 3 | 1.9 |
| capillary refill time | 156 | 3 | 1.9 |
| pain medication | 158 | 1 | 0.6 |
| vena cava diameter mm | 92 | 67 | 42.1 |

## Supplementary Table S6. Complete-case sensitivity analysis

Each endpoint restricted to assessments in which every component of its label was observed. For pain this leaves the dataset unchanged, because no VAS score used in the analysis was imputed. For dehydration the restriction is substantial, mainly because it requires an inferior vena cava measurement. Values are the mean across five random seeds.

| **Endpoint** | **Windows, all** | **Windows, complete case** | **Folds** | **Mean per-participant AUC (range)** | **Pooled AUC** |
| --- | --- | --- | --- | --- | --- |
| Pain | 1,414 | 1,414 | 8 | 0.557 (0.533-0.599) | 0.736 |
| Dehydration | 1,414 | 806 | 9 | 0.535 (0.505-0.561) | 0.481 |

The conclusions are unchanged for both endpoints.

## Supplementary figure legends

Supplementary Figures S1–S2 (combined). Held-out ROC curve for every evaluated participant. (A) pain, (B) dehydration. Legend entries give each participant's AUC and the number of positive and negative windows contributed; the heavy line is the mean across participants.

Supplementary Figure S4. Analysis pipeline of the VIENNESE study, from continuous smartwatch recording and thrice-weekly clinical assessment through window aggregation, feature engineering and the participant-level data-quality filter, to nested leave-one-patient-out cross-validation and evaluation.

Supplementary Figure S3. Averaged held-out ROC curve for the exploratory female-only pain analysis across 7 LOPOCV folds. With seven participants and no evaluable male subgroup, no inference is drawn from this comparison.
